# Guava Leaf Essential Oil as a Potent Antioxidant and Anticancer Agent: Validated through Experimental and Computational Study

**DOI:** 10.3390/antiox11112204

**Published:** 2022-11-07

**Authors:** Ashok Kumar Mandal, Samrat Paudel, Anisha Pandey, Parasmani Yadav, Prateek Pathak, Maria Grishina, Mariusz Jaremko, Abdul-Hamid Emwas, Habibullah Khalilullah, Amita Verma

**Affiliations:** 1 Natural Product Research Laboratory, Thapathali, Kathmandu 44600, Nepal; 2 Bioorganic and Medicinal Chemistry Research Laboratory, Department of Pharmaceutical Sciences, Sam Higginbottom University of Agriculture, Technology and Sciences, Prayagraj 211007, India; 3 Department of Biotechnology, Kathmandu University, Dhulikhel 45200, Nepal; 4 Department of Biotechnology, National College, Tribhuvan University, Naya Bazar, Kathmandu 44600, Nepal; 5 Laboratory of Computational Modeling of Drugs, Higher Medical and Biological School, South Ural State University, Chelyabinsk 454008, Russia; 6 Smart-Health Initiative (SHI) and Red Sea Research Center (RSRC), Division of Biological and Environmental Sciences and Engineering (BESE), King Abdullah University of Science and Technology (KAUST), Thuwal 23955-6900, Saudi Arabia; 7 Core Labs, King Abdullah University of Science and Technology (KAUST), Thuwal 23955-6900, Saudi Arabia; 8 Department of Pharmaceutical Chemistry and Pharmacognosy, Unaizah College of Pharmacy, Qassim University, Unaizah 51911, Saudi Arabia

**Keywords:** *Psidium guajava* L., essential oil, GC-MS, antioxidant activity, cancer, molecular docking

## Abstract

Several drugs now employed in cancer therapy were discovered as a result of anticancer drug research based on natural products. Here, we reported the in vitro antioxidant and anticancer activity followed by in silico anticancer and estrogen-like activity of *Psidium guajava* L. essential oil against ER-α receptors which lead to potential inhibitory action against breast cancer pathways. Methods: The bioactive compounds in guava essential oil were screened using gas chromatography–mass spectrometry (GC-MS). Similarly, the antioxidant properties of the extracted oil were evaluated using 2,2-Diphenyl-1-picrylhydrazyl scavenging assay. Furthermore, the in vitro anticancer activity of guava oil was observed through the MTT assay and an in silico molecular docking experiment was also carried out to ensure that they fit into the estrogen receptors (ERs) and possess anticancer potential. Results: The GC–MS profile of the essential oil revealed the presence of 17 chemicals, with limonene (51.3%), eucalyptol (21.3%), caryophyllene oxide (6.2%), caryophyllene (5.6%), and nerolidol (4.5%) occupying more than one-third of the chromatographic spectrum zone. Guava leaves’ essential oil (EO) inhibited DPPH (2,2-diphenyl-1-picrylhydrazyl) radicals and exhibited concentration dependent free radical scavenging activity, acting as a potent antioxidant with an IC_50_ value of 29.3 ± 0.67 µg/mL. The outcome of the MTT assay showed that the extracted guava oil had nearly the same efficacy against breast and liver cancer cells at a low concentration (1 µg/mL), giving 98.3 ± 0.3% and 98.5 ± 0.4% cell viability against HepG2 at 1 µg/mL, respectively. When the concentration of essential oil was increased, it showed a small reduction in the percentage of viable cells. While conducting an in silico study of all the screened compounds, the potential for hydroxycaryophyllene, caryophyllene, caryophyllene oxide, humulene, terpineol, and calamenene to inhibit tumor growth was bolstered due to a resemblance to 4-hydroxytamoxifen, thereby implying that these compounds may act as selective estrogen receptor modulators (SERMs). The ADME analysis of the compounds indicated above revealed that they exhibit excellent drug likeness properties and follow the Lipinski rule of five. Conclusions: Consequently, they have a substantial anticancer therapeutic potential and can be used for novel drug discovery in the effort to minimize the global burden of breast cancer.

## 1. Introduction

It is obvious that traditional medicine has always played an important role in the primary health care of people, especially in developing countries. *Psidium guajava* (*P. guajava*) (family Myrtaceae), commonly known as guava, is a medicinal tree native to Central America and cultivated mainly in tropical and subtropical regions of the world [1]. It can grow at an altitude of about 1500 m above sea level with an annual rainfall of less than 1000 mm [2]. This widespread tropical plant belongs to the Myrtaceae family and is one of the most common fruit crops in the Nepalese Terai, inner Terai, and mountainous regions due to its high value, medicinal properties, and income potential [3]. In addition to its high nutritional value the plant is also associated with a long history of traditional use. Hundreds of years ago the guava plant was valued not only for its delicious fruit but also for the various parts of the plant which are important in folk medicine. Since ancient times the tree has been valued for its therapeutic properties which range from antibacterial/antifungal activities to anticancer properties [4]. It has been documented that the decoction prepared from guava leaves was eaten daily to eliminate problems such as painful menstruation, miscarriage, and uterine bleeding. Additionally, it was used to treat lung and stomach cancer [5,6].

Essential oils are basically defined as the predominantly volatile component of a plant, separated by a physical process, which has the odor and other characteristic properties of that plant. Extraction of essential oils can be accomplished through various techniques, such as hydrodistillation, steam distillation, hydrodiffusion, or even by using solvents [7]. Traditionally, essential oils have been used to treat insomnia, convulsions, epilepsy, bronchitis, asthma attacks, wounds, pain, obesity, and diabetes mellitus. Nowadays, essential oils are also greatly popular due to their use in the treatment of microbial diseases as well as their anticancer, antioxidant, and anti-inflammatory properties [1].

The essential oil extracted from guava leaves, referred to in this text as guava leaf essential oil (GLEO), contains resin, tannins, flavonoids, triterpenes, malic acid, eugenol, cineole, and fat. Due to the presence of these compounds, guava leaves are suitable for various medicinal uses such as the treatment of diabetes, hypertension, diarrhea, respiratory diseases, obesity, fever reduction, wound healing, anti-inflammation, and pain relief [8]. The presence of compounds such as phenols, terpenes, terpenoids, quercetin, glycosides, acetic acid, protocatechuic acid, citric acid, glutamic acid, malonic acid, cis-aconitic acid, trans-aconitic acid, epicatechin, asparagine, and xanthine make it an excellent antioxidant [9]. Guava leaves have shown inhibitory effects on several cancer cell lines, such as breast cancer [10] (MCF-7 and MDA-MB-231), prostate cancer [11] (PC-3, DU 145 and LNCaP), cervical cancer [12] (HeLa), colon cancer [13] (COLO320DM), and nasopharyngeal cancer [14] (KB).

The hexane fraction of guava leaves has been shown to inhibit the AKT/mTOR/S6K1 pathway, which is related to tumorigenesis, angiogenesis, and metastasis [15,16]. In vitro, in vivo, and in silico studies revealed anticancer and estrogenic effects of *Psidium guavaja* L. (guava), as evidenced by the compounds meroterpenes, guaial, psidial A, psiguadial A, and psiguadial B, respectively [17].

Estrogen receptors which are found in endometrial cells, breast cancer cells, and ovarian stromal cells, are responsible for controlling proliferation, maturation, metabolism, differentiation, homeostasis, inflammation, and apoptosis in breast cancer. The estrogen receptors are activated as a result of the binding of a steroidal ligand-17β-estradiol, commonly known as estrogen, to a cell.

Estrogen receptors alpha (ERα) and beta (ERβ) are mainly found in humans. ERα is most expressed in the uterus and mammary glands. Studies illustrated that in 70% of human breast cancers cases ERα was found to be overexpressed. It has also been clinically proven that ERα plays an important role in breast cancer [18,19,20]; therefore, selective estrogen receptor modules (SERMs) such as tamoxifen and raloxifene are commonly used in the treatment of breast and other related cancers.

It is also evident that breast cancers develop resistances to SERMs [21,22] after a certain period of exposure. Therefore, the continuous search for new ERα inhibitors in anticancer drug discovery is extremely worthwhile. In silico modeling allows for the screening of small molecules, called ligands that can target larger macromolecules such as proteins. The affinity of such small molecules for the active site of a protein can be predicted computationally based on binding energy. The compounds exhibit strong and stable interactions allowing them to be considered as potential drug candidates. In recent years, the investigation of potential drug molecules using computational modeling has increased due to its fast, inexpensive, and effective technology.

In this research, GLEO was tested for antioxidant potency and in silico docking studies were performed on the compounds detected by GC–MS to assess their affinity on ER-α receptors, which leads to the potential inhibitory action towards the breast cancer pathway.

## 2. Materials and Methods

### 2.1. Sample Collection and Extraction of Essential Oil

Samples of *P. guajava* leaves were collected in Kathmandu, Nepal (27°40′58.3284″ N, 85°19′45.5376″ E). The collected specimens were authenticated by a botanist at the Natural Product Research Laboratory, Thapathali, Kathmandu. The voucher specimen was deposited in the National Herbarium and Botanical Laboratory (KATH), Godawari, Lalitpur, Nepal.

The air-dried and crushed leaves were cut and then hydrodistilled for 3 h in a Clevenger-type apparatus. The obtained essential oil was dried over anhydrous sodium sulfate and stored in sealed vials in a refrigerator at 4 °C until further analysis was performed.

### 2.2. Gas Chromatography-Mass Spectrometry (GC-MS) Analysis of Essential Oil

The composition of the essential oil extracted from the leaves of *P. guajava* (GLEO) was determined using a gas chromatography-mass spectrometer (GCMS) (Shimadzu GC-2010 Plus, Shimadzu, Kyoto, Japan) in conjunction with a capillary column RTX-5 MS with dimensions 60 m × 0.25 mm × 0.25 μm. Helium was used as the carrier gas with a flow rate of 106.1 mL/min at a pressure of 23.3 Pa. The injector was set at 250 °C and the operating temperature was 40–230 °C (3 °C/min), with the split ratio set at 150:1. The detector voltage was adjusted depending on the tuning result. The sample was diluted with hexane and 1 μL of the diluted sample was injected into the gas chromatography (GC) column at a column oven temperature of 40 °C. The constituents of the EO were identified by comparing the mass spectra with NIST and the FFNSC 4 mass spectra library [23] of the GC–MS data system and confirmed by comparing their Kovates retention indices (KI) with the reported masses in the range 40–350 [24].

### 2.3. Determination of Antioxidant Activity

Antioxidant activity was determined using the assay proposed by Mensor et al. to capture free 2,2-diphenyl-1-picrylhydrazyl (DPPH) radicals [25]. 100 µL of the plant extract at different concentrations in 50% (*v*/*v*) dimethyl sulfoxide (DMSO) (125 to 3.9 µg/mL) were mixed with 100 µL of DPPH solution (0.01 M) in methanol. The reaction mixture was shaken and allowed to react in the dark for 30 min. Finally, the absorbance was measured at 517 nm in a microplate reader (EpochTM 2 microplate spectrophotometer, BioTek Instruments, Winooski, VT, USA). Quercetin solution in methanol (1.25–20 µg/mL) was used as an antioxidant standard and 50% (*v*/*v*) DMSO was used as a negative control. The entire experiment was repeated in triplicate. Free radical scavenging activity was calculated using the following formula.

$$\%\;\mathrm{inhibition}=\left(\frac{A_{\mathrm{control}}-A_{\mathrm{sample}}}{A_{\mathrm{control}}}\right)\times 100$$

where, *A*_control_ is the absorbance of the control and *A*_sample_ is the absorbance of the sample. 

### 2.4. In-Vitro Anticancer Activity Assay

The anticancer activity of the extracted oil from the leaves of *P. guajava* was studied using the MTT test. For this purpose, the extracted essential oil (at different concentrations) was diluted into the culture medium, added to the wells, and incubated for 48 h. Then MTT (3-(4,5-dimethylthiazol-2-yl)-2,5-diphenyltetrazolium bromide) (5 mg/mL) was added to each well and incubated for another 4 h. At the end of the incubation period the supernatant was removed from each well of the plates and DMSO was added to dissolve the formazan crystals. The absorbance of the samples and the blank were measured at 540 nm, and the value of the blank was not considered for measuring the actual absorbance of the test samples. The entire experiment was repeated in triplicate [26,27,28] and the percentage cell viability was calculated using following formula:

$$\mathrm{Cell}\;\mathrm{viability}\;\left(\%\right)=\frac{\mathrm{Mean}\;\mathrm{OD}\;\left(\mathrm{Sample}\right)}{\mathrm{Mean}\;\mathrm{OD}\;\left(\mathrm{Control}\right)}\times 100$$

### 2.5. In-Silico Modelling for Potential Anti-Cancer Constituents

**Figure 1:**
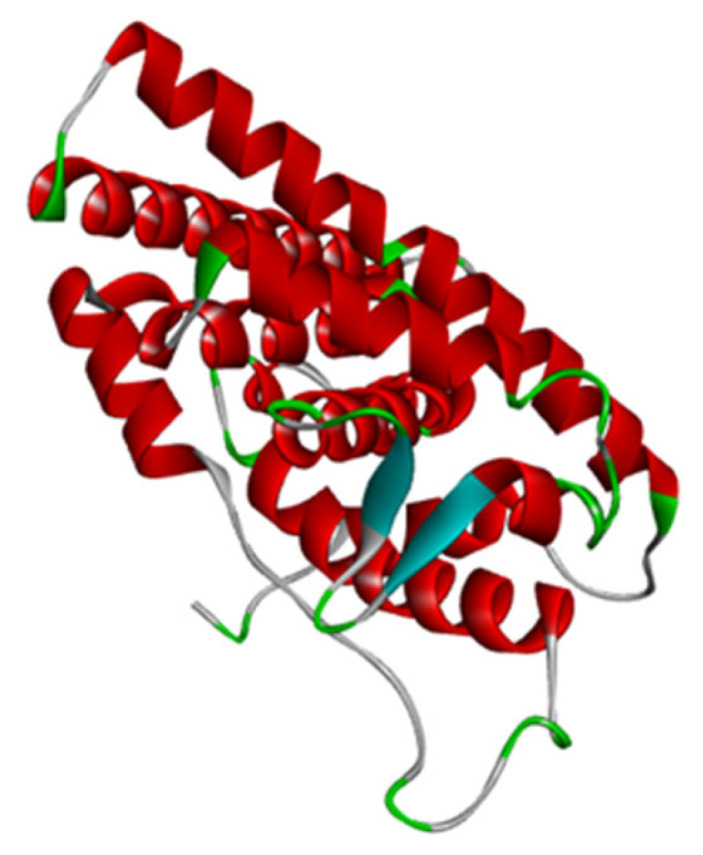
The structure of the human receptor alpha protein (3ERT) extracted from the protein data bank after removing water molecules and cocrystallized ligand molecules.

The 3D structure of the human estrogen receptor alpha protein (PDB id: 3ERT) [29] in complex with 4-hydroxytamoxifen submitted by Shiau et al. [29] was downloaded from the protein data bank (PDB) (https://www.rcsb.org/, accessed on 20 August 2022) (Figure 1). The crystal structure was generated using an X-ray diffraction method with a resolution of 1.90 Å. Furthermore, the native ligand 4-hydroxytamoxifen was separated and used as a ligand for re-docking. 3D structures of the tested compounds (detected from the GC–MS) were downloaded from PubChem (https://pubchem.ncbi.nlm.nih.gov, accessed on 20 August 2022). Additionally, AutoDock Tools 4 [30] was used for the preparation of the docking files and AutoDock Vina [31] was used for molecular docking. The docked complexes were visualized in Discovery Studio visualizer (BIOVIA, Dassault Systèmes, Discovery Studio Visualizer, 4.5, Dassault Systèmes, San Diego, CA, USA).

#### 2.5.1. Preparation of Receptors and Ligands

The cocrystallized ligand 4-hydroxytamoxifen was removed from the pdb file for 3ERT using PyMOL 2 [32] and saved in a .pdb format. The missing residues in proteins were added and the energy was minimized using DeepView (http://www.expasy.org/spdbv/, accessed on 21 August 2022) [33]. The energy minimized pdb file was opened in AutoDock Tool 4 [34] for further processing. Water molecules were removed, and polar hydrogens were added to the protein. The macromolecule was checked for the missing atoms and was repaired. The appropriate Kollman charges were added to the protein and saved as a .pdbqt file after assigning the AD4 atom type [35].

Similarly, the ligand file was converted to .pdb format from .sdf format using Open Babel (http://www.openbabel.org, accessed on 21 August 2022) [36]. Further, Gasteiger charge was computed and the root for the torsion tree was detected using AutoDock 4. After choosing the torsion and setting the number of torsions the files was saved in pdbqt format. The same action was repeated for all the ligand files.

#### 2.5.2. Docking Using AutoDock Vina

The dockings between the macromolecule and the ligands were performed using Auto Dock Vina tool [31]. The active site of the 3ERT was determined by taking from the native hydroxytamoxifen, cocrystallized inhibitor. The centers for docking were 29.944, −1.861 and 24.611 (center_*x*, center_*y*, center_*z*) with spacing 18, 14 and 18 (size_*x*, size_*y*, size_*z*) on the *X*, *Y* and *Z* axes.

#### 2.5.3. Analysis of Docking Result

The docking results were analyzed on the basis of the binding score. Discovery Studio 2021 was used for the visualization of the interaction between the macromolecule and the ligands. The configurations with the lowest energy of binding were chosen for the visualization and further analysis. The interaction between the hydroxytamoxifen and the 3ERT obtained by docking was compared to the interaction shown in the protein data bank. For the study of amino acid interaction between ligands and the macromolecule, only the ligands with binding affinity equal to or less than −8 kcal/mol were taken.

### 2.6. Complementarity Assessment Using AlteQ Orbit-Free Quantum Chemical Method

In order to make a more detailed selection of the structures obtained using the docking method, we used a recently proposed approach that assesses the complementarity of the electronic structures between the enzyme and ligand. This approach is based on the AlteQ orbit-free quantum chemical method and is described in detail in the literature [37,38,39]. In short, in the case of high complementarity between the electronic structures of the enzyme and ligand, a high correlation between the sum of the distances SUMRLRE and the complementarity factor ($CF_{1}$), which is determined at a point *m* in the intermolecular space and dependent on the electron density supplied by the enzyme and ligand to this point, should be observed.

$$CF_{1}=a_{\mathrm{CF}1}+b_{\mathrm{CF}1}\times \mathrm{SUMRLRE}\;\mathrm{SUMRLRE}=R_{\mathrm{ml}}+R_{\mathrm{me}}$$

where $R_{\mathrm{ml}}$ is the distance between the *m*-th point in space and the *l*-th ligand’s atom with the greatest contribution to the ligand’s electron density at that point. $R_{\mathrm{me}}$ is, analogously, the distance between the *m*-th point and *e*-th enzyme’s atom with the greatest contribution to the enzyme’s electron density at that point. $a_{\mathrm{CFk}}$ and $b_{\mathrm{CFk}}$ are parameters of the equation.

$$CF_{1}=\ln\left(\frac{\rho_{E}\times \rho_{e\left(\mathrm{CNT}\right)}}{N_{e}}\right)+\ln\left(\frac{\rho_{L}\times \rho_{l\left(\mathrm{CNT}\right)}}{N_{l}}\right)$$

where $\rho_{E}$ and $\rho_{L}$ are the outer shells’ electron densities of the enzyme and the ligand at the *m*-th point (in e/Å^3^); $\rho_{e\left(\mathrm{CNT}\right)}$ and $\rho_{l\left(\mathrm{CNT}\right)}$ are the electron densities at the centers of the *e*-th enzyme atom and the *l*-th ligand atom, respectively; $N_{e}$ and $N_{l}$ are the atomic numbers of the *e*-th enzyme atom and the *l*-th ligand atom, respectively.

### 2.7. ADME Analysis

The drug likenesses of the ligands were tested on the basis of the Lipinski Rule of 5. The parameters taken as per the Lipinski rule are molecular weight, lipophilicity (MLOGP), number of hydrogen bond acceptors, number of hydrogen bond donors, and molar refractivity [40]. The ligand files in the .sdf format obtained from PubChem were entered in the SwissADME individually [41] (http://www.swissadme.ch, accessed on 23 August 2022) and the values of the 5-Lipinski parameter were analyzed.

## 3. Results and Discussion

### 3.1. GC–MS Analysis of Essential Oil

**Figure 2:**
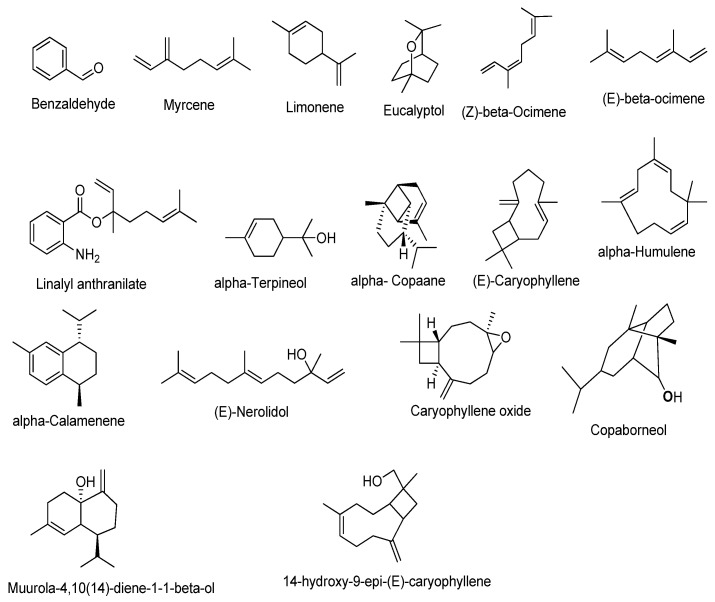
The structure of the compounds obtained after GC–MS analysis. The common name of each compound is given below each compound.

GC–MS analysis of the essential oil detected the presence of 17 major compounds, which have been listed in Table 1 and the structures were drawn using ChemDraw Ultra, Version 12.0.2.1076 (Figure 2). Constituent concentration in the tested oil was directly calculated from their peak area. GC–MS analysis displayed that extracted oil primarily contains limonene (51.3%) and eucalyptol (21.3%). Apart from these, some other phytocompounds such as caryophyllene oxide (6.2%), caryophyllene (5.6%), and nerolidol (4.5%) were also found in lower concentrations. A similar pattern of volatile component composition was reported in the essential oil of guava leaves in a study conducted by Satyal et al. (2015) [42].

The major constituent Limonene is well established as a safe anticancer agent against various forms of carcinogenesis [43]. Evidence from the clinical trials (NCT01046929, NCT01459172) revealed its effectiveness against breast cancer which has established limonene as a potent breast cancer preventive agent. Notably, the major compounds revealed in guava leaf essential oils have been shown to have strong anticancer potential.

### 3.2. Antioxidant Activity of Essential Oil

**Figure 3:**
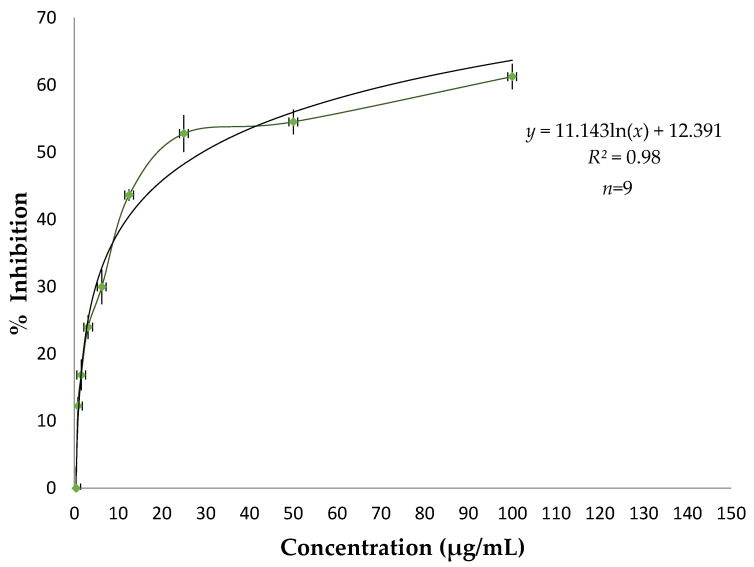
Antioxidant property (IC_50_) of GLEO along with its logarithmic equation and *R^2^* value.

The antioxidant property of GLEO was determined using DPPH-scavenging assay and compared with quercetin as the standard. The secondary metabolites present in the GLEO were found to have the ability to donate electrons, turning the purple color of DPPH to yellow. The IC_50_ value is the concentration at which the initial concentration of DPPH is minimized by 50% and is used for the calculation of antioxidant potency (Illustrated in Figure 3). The lower the IC_50_ value, the greater the antioxidant potency of the essential oil. The DPPH-scavenging was found to be concentration dependent with 24 ± 2%, 30 ± 3%, 44 ± 1%, 53 ± 3%, 55 ± 2%, and 61 ± 2% inhibition at concentrations of 3.1, 6.3, 12.5, 25, 50, and 100 µg/mL, respectively. The statistical calculation for the IC_50_ value was performed and found to be 29.3 ± 0.7 µg/mL, which is higher than that of standard quercetin (IC_50_ value:5.23 ± 0.38 µg/mL). The standard quercetin has an IC_50_ value 5.23 ± 0.38 µg/mL. The primary content of monoterpenes like limonene and eucalyptol act as a radical scavenger through an electron or hydrogen donating mechanism and the potential for antioxidant activity was shown to be closely correlated with the antioxidant activity previously reported [44]. As part of comparative study, we found results similar to those obtained by Zang et al. (IC_50_ of 17.66 ± 0.07 µg/mL) [45].

### 3.3. In Vitro Anticancer Activity

In vitro anticancer activity of oils extracted from *P. guajava* leaves was evaluated through the MTT assay against liver and breast cancer cell lines. In vitro results demonstrated that the oils extracted from *P. guajava* leaves hold mild to significant inhibitory potential (Table 2). Briefly, *P. guajava* oils displayed 98.3 ± 0.3% and 98.5 ± 0.4% cell viability against HepG2 at 1 µg/mL, respectively. The results indicated that at lower concentrations (1 µg/mL) the extracted oil has similar efficacy against breast and hepatic cancer cells. Furthermore, a minor upsurge in the concentration of oils extracted from *P. guajava* leaves (at 2 and 10 µg/mL) demonstrated slight downregulation in the percentage of cell viability (97.2 ± 0.4%, 89.3 ± 0.5% cell viability against HepG2 and 96.2 ± 0.3%, 89.5 ± 0.3% cell viability against MCF-7, respectively). At 25, 50, 75, 100, 200, and 250 µg/mL concentrations the oils extracted from *P. guajava* leaves also illustrated a pattern of continuous incremental change in inhibitory activity (82.3 ± 0.4%, 78.3± 0.2%, 72.1 ± 0.3%, 68.3 ± 0.5%, 62.2 ± 0.5%, and 54.7 ± 0.3% cell viability against HepG2; 84.3 ± 0.3%, 78.7 ± 0.3%, 72.4 ± 0.2%, 67.3 ± 0.4%, 58.3 ± 0.4%, and 52.6 ± 0.3% cell viability against MCF-7, respectively). These results clearly establish the benefit of the extracted oils from *P. guajava* leaves. To study potential toxicity, the extracted oil was evaluated over 12A normal cells (normal breast cells). The toxicity and specificity studies demonstrated that the plants have only minor effects on normal cell lines and can be allowed for further application.

### 3.4. In Silico Modelling for Potential AntiCancer Constituents

**Figure 4:**
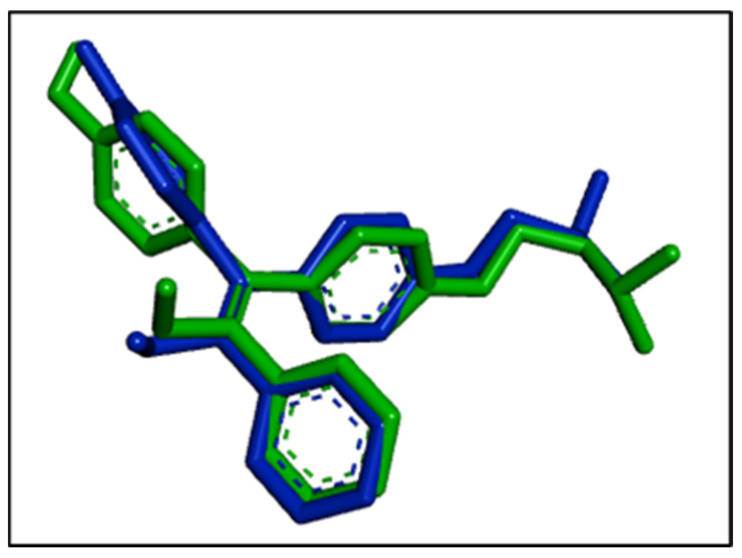
The superimposition in the binding position of the cocrystallized hydroxytamoxifen (blue colored) and computationally obtained binding position of hydroxytamoxifen (green colored).

Docking analysis allows the prediction of position, orientation, and conformation of ligands in the binding site of the larger macromolecule [46]. The reference ligand, hydroxytamoxifen, was also included in the docking analysis to validate the docking procedure. Among the eight conformations of hydroxytamoxifen obtained from docking, the conformation with the lowest binding affinity (−9.7 kcal/mol) was superimposed with the experimental conformation, thereby validating our docking methodology. The interaction between the hydroxytamoxifen and the 3ERT obtained from docking was graphically compared with the interaction provided in the protein data bank. Both the interactions were superimposed with slight changes in the terminal regions. The superimposition is shown in Figure 4.

The reference ligand hydroxytamoxifen was found to have the greatest binding score with an affinity of −9.7 kcal/mol. Alternatively, the docking analysis using AutoDock Vina revealed that caryophyllene and caryophyllene oxide have the highest affinity for the ER among all the compounds in guava essential oil. Caryophyllene and caryophyllene oxide are natural bicyclic sesquiterpenes, which have been experimentally proven to have anticancer properties in vitro [47]. Humulene which showed an affinity of −8.3 kcal/mol has also been proven to exhibit anticancer properties in vitro [48,49]. The anticancer properties of muurola-4,10(14)-dien-8beta-ol, copaborneol, and copaene against breast cancer has been less studied in vitro. Since our molecular docking analysis clearly showed a strong affinity between these compounds and estrogen receptors of choice, these compounds can be further explored as potential anticancer agents. The binding affinity for all the compounds has been presented in Table 3.

**Figure 5:**
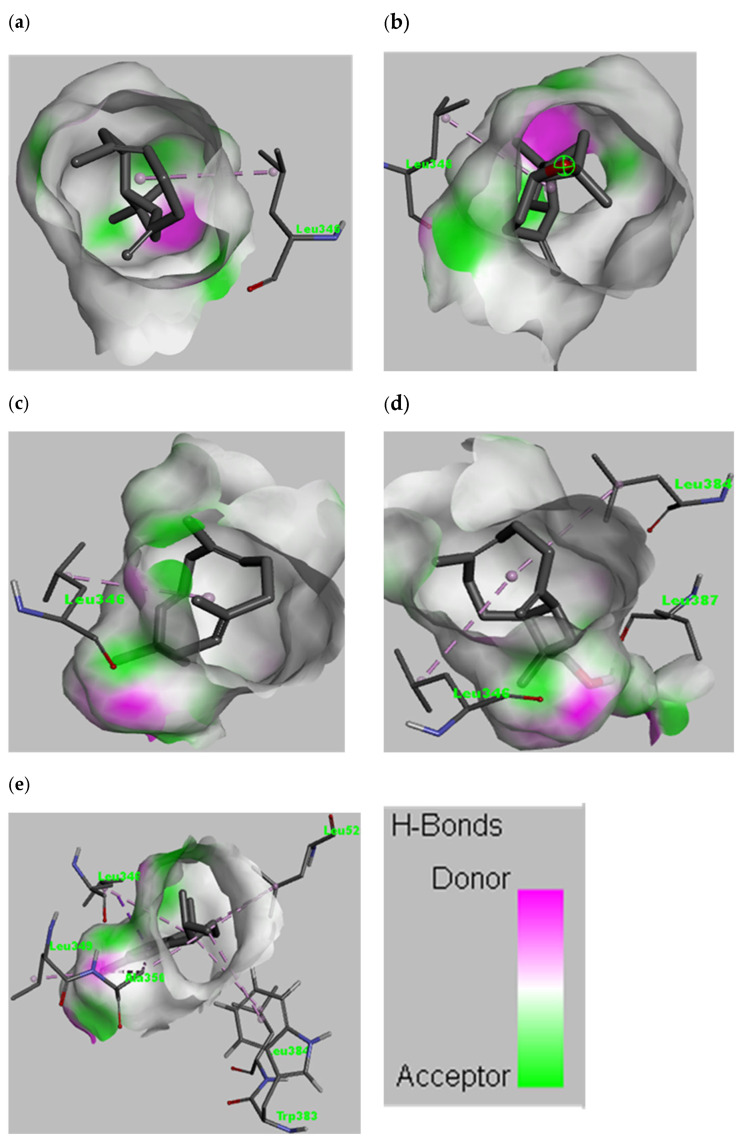
Interactions between the protein molecule and ligands shown in a 3D representation. Only interacting atoms are shown. (**a**) cryophyllene, (**b**) cryophyllene oxide, (**c**) humulene, (**d**) 14-hydroxy-9-epi-(E)-caryophyllene, and (**e**) calamenene. The H bond donor and acceptor tendency is given in color index.

**Figure 6:**
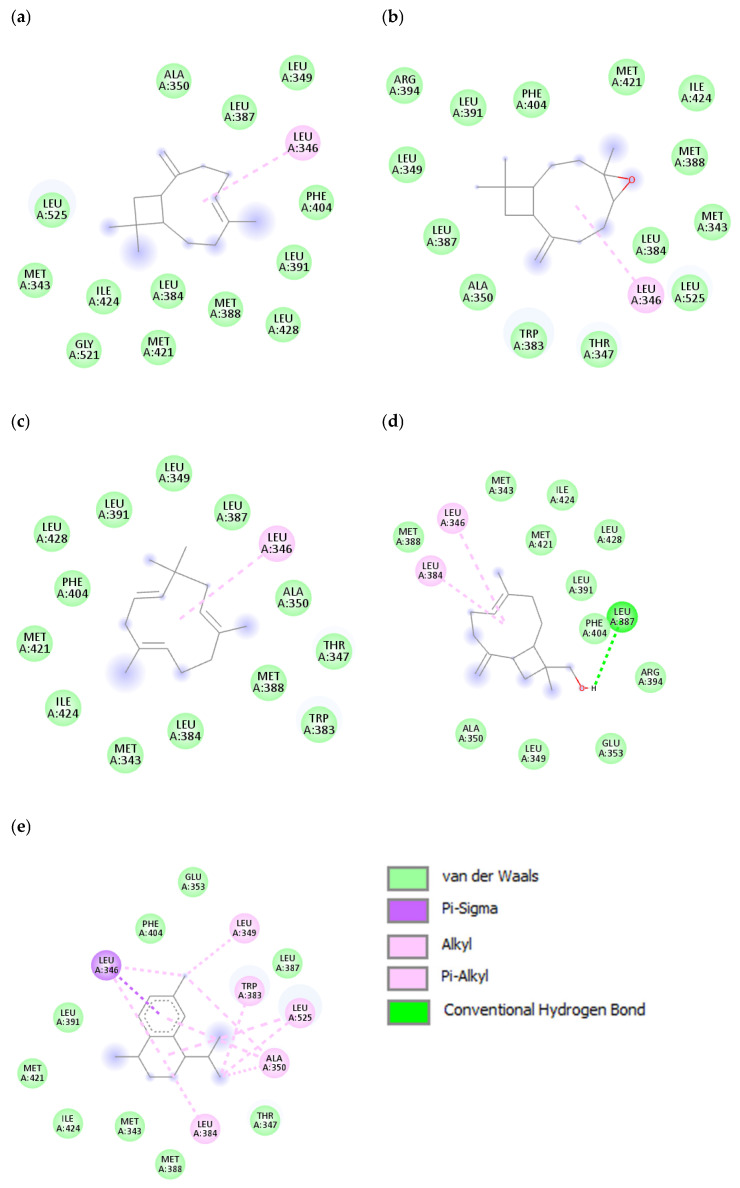
Interactions between the protein molecule and ligands shown in a 2D representation. Interacting atoms as well as pocket atoms are shown (**a**) cryophyllene, (**b**) cryophyllene oxide, (**c**) humulene, (**d**) 14-hydroxy-9-epi-(E)-caryophyllene, and (**e**) calamenene. The legend for the interactions involved is given in the left lower box.

The interactions between selected ligands with ER-α are shown in Figure 5 and Figure 6. The five ligands with an affinity less than −8.0 kcal/mol were selected for graphical illustration. The amino acid responsible for the interaction was Leu346 for most of the ligands. Moreover, amino acids such as Leu384, Leu387, Leu349, Ala350, Trp383, and Leu525 are involved in the interaction. Different types of interactions such as van der Waals, alkyl, Pi-alkyl, Pi-sigma and conventional hydrogen bond are present with the van der Waals and the alkyl interactions being the most common. The amino acids involved in the interaction of selected ligands are presented in Table 4.

### 3.5. Complementarity Analysis of the Electronic Structures of Enzyme and Ligand

It was found that for experimentally discovered structures: the square of the correlation coefficient Rcor2 does not fall below 0.81; the maximum value of the complementarity factor MAX (CF1) characterizing the efficiency of enzyme-ligand overlaps should be −5÷−2; and the parameters of the linear equation should be as close to the experimental complex as possible because interaction with the site is mainly due to similar overlaps involving the same amino acid residues. These principles were used to select the most correct structures obtained through docking. Docked complexes of calamenene (model 18) and muurola-4,10(14)-diene-1-1-beta-ol (model 3) demonstrated the best complementarity among all docked complexes (Table 5).

### 3.6. ADME Analysis

The ligands showing an affinity less than −8 kcal/mol were studied for druglikeness properties using Lipinski’s rule of 5. Lipinski’s rule considers molecular weight, lipophilicity, number of H bond donors and acceptors, as well as molar refractivity of the compound for the evaluation of the druglikeness of a compound. Among the 15 ligands, the analysis of the five compounds was performed for ADME study and the analysis revealed that all the compounds pass druglikeness tests. Compounds cryophyllene oxide and 14-Hydroxy-9-epi-(E)-caryophyllene showed zero violation while the compounds cryophyllene, humulene, and calamenene showed one violation (MLOGP > 4.15) of the Lipinski test. The computation ADME predictions of the ligands are illustrated in Table 6.

### 3.7. Probable Mechanism of Action

**Figure 7:**
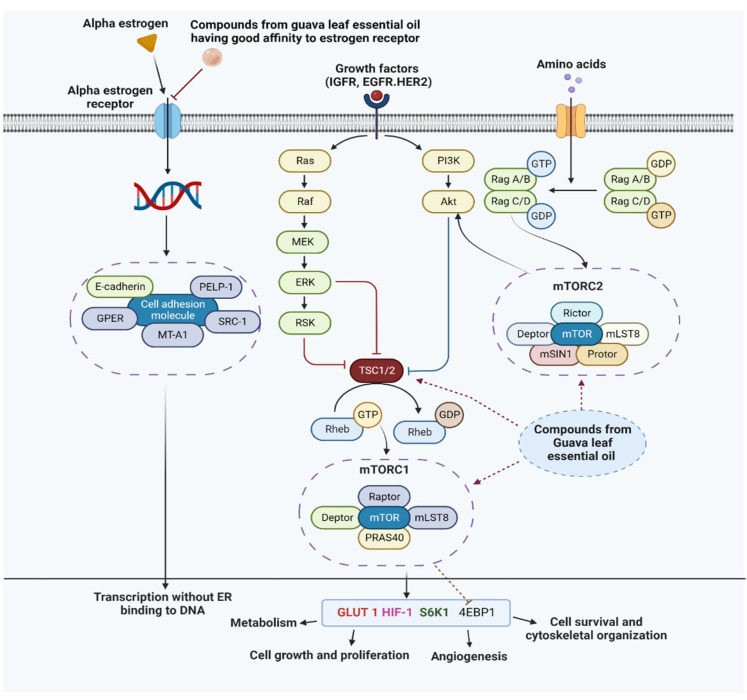
An illustrative approach to the mechanism of action of compounds from guava leaves essential oil against estrogen receptor. RTKs: Receptor tyrosine kinase; EGFR: Epidermal growth factor receptor; HER2: Human epidermal growth factor receptor 2; IGFR: Insulin-like growth factor receptor; SRC: Steroid receptor co-activator; pi3K: Phosphoinositide 3-kinase; ERK: Extracellular signal regulated kinase; PELP: Proline, Glutamate and Leucine Rich Protein; MT A1: Metastasis Associated Protein-1; GPER: G-protein coupled estrogen receptor; TSC: Tuberous sclerosis; 4EBP1: Eukaryotic initiation factor 4E binding protein 1; PTEN: Phosphatase and tensin homolog; mTORC1/2: Mammalian target of rapamycin complex 1/2; HIF-1: Hypoxia inducible factor 1; Akt: protein kinase B; Glut1: Glucose transporter 1; GTPase: Guanosine triphosphatase; IRS: Insulin receptor substrate; PI3K: Phosphatidylinositol 3 kinase; S6K1: S6 kinase 1. (The Figure was generated using Bio-render).

The discovery of molecular biomarkers is vital considering breast cancer is a very diverse disease. The PI3K/Akt/mTOR signaling pathway is a significant intracellular pathway that promotes cell proliferation in breast cancer. Tuberous sclerosis (TSC), which acts as a GTPase activating protein for Rheb, is inhibited by the activation of Akt. Phenomenologically it stimulates mTORC1 to undergo anabolic growth of its cells via its action on S6K1 and 4EBP1, which consequently promotes protein synthesis, metabolism, and cell proliferation [50,51]. There is credible evidence that caryophyllene and caryophyllene oxide have a significant role in inhibiting the PI3K/Akt/mTOR signaling pathway, thereby asserting anticancer potency [52]. Moreover, the volatile compounds from the GLEO are supposed to exhibit the mechanism of action followed by selective estrogen receptor modulators (SERM) to inhibit the proliferation of breast cancer cells. The molecular signaling pathway of estrogen and estrogen receptor-like ligand activation and competitive inhibition, protein–protein interaction with other transcription factors for the regulation of genes through indirect binding following serum response element transcription activation (specificity protein 1 and activator protein 1), ligand dependent activation of various signaling pathways which regulates the multiple mediators supporting transcription without ER binding to DNA [53]. An overview of the mechanistic approach which is supposed to be exhibited by the compounds from GLEO is illustrated in Figure 7.

## 4. Conclusions

In this article, we elucidated the composition of the essential oil of guava leaves in order to explore its antioxidant properties and anticancer potency. The GC–MS analysis of volatile oils revealed the presence of various anticancer compounds implying a promising DPPH-Scavenging potency. The in vitro anticancer test accomplished using an MTT assay showed a promising result that indicated guava essential oil may work against hepatic and breast cancer cells at different concentrations. Similarly, the in silico study on anticancer activity revealed the high affinity of our reference ligand (hydroxytamoxifen) towards the receptor (α-ER). Since, α-ER is primarily responsible for the progression of breast cancer and since hydroxytamoxifen, a metabolite of hormonal drug tamoxifen, showcased an excellent binding affinity with the receptor, our ligand may be beneficial as it inhibits the estrogen from binding with estrogen receptors, thereby inhibiting cancer cell proliferation. The compounds shown to have significant docking scores were found to have an excellent druglikeness property (ADME) as they pass the Lipinski’s rule of 5 test. With all these results, we can conclude that the volatile compounds in GLEO selectively bind to α-ER receptors and could be potential therapeutics for estrogen dependent antiproliferative activity.

## Figures and Tables

**Table 1:** Different compounds detected from GC–MS with their retention time.

Peak Number	Retention Time (min)	Compound	Area	Area Percentage
1	14.5	Benzaldehyde	52,096	0.5
2	15.7	Myrcene	63,902	0.6
3	17.6	Limonene	5,248,363	51.3
4	17.8	Eucalyptol	2,177,857	21.3
5	17.9	Ocimene <(Z)-, beta->	135,011	1.3
6	18.4	Ocimene <(E)-, beta->	61,578	0.6
7	20.9	Linalyl anthranilate	83,586	0.8
8	25.4	Terpineol <alpha->	129,922	1.3
9	34.0	Copaane <alpha->	110,063	1.1
10	36.0	Caryophyllene <(E)->	572,209	5.6
11	37.4	Humulene <alpha->	72,787	0.7
12	40.2	Calamenene <alpha->	88,207	0.9
13	41.5	Nerolidol <(E)->	460,010	4.5
14	42.8	Caryophyllene oxide	637,587	6.2
15	43.6	Copaborneol	90,736	0.9
16	44.4	Muurola-4,10(14)-diene-1-1-beta-ol	119,809	1.2
17	44.8	Caryophyllene <14-hydroxy-9-epi-(E)->	131,421	1.3

**Table 2:** Percentage viability of cancer cell lines of the oils extracted from *P. guajava* leaves at different concentrations.

Source	Concentration (µg/mL)	Percentage Viability					
		Cancer Cell Lines			Normal Cell Line		
		HepG-2		MCF-7		MCF-12A	
Control	-	0		0		0	
Oil extracted from *P. guajava* leaves	1	98.3 ± 0.3		98.5 ± 0.4		99.5 ± 0.2	
	2	97.2 ± 0.4		96.2 ± 0.3		97.1 ± 0.3	
	10	89.3 ± 0.5		89.5 ± 0.3		93.2 ± 0.5	
	25	82.3 ± 0.4		84.3 ± 0.3		91.2 ± 0.5	
	50	78.3 ± 0.2		78.7 ± 0.3		90.1 ± 0.3	
	75	72.1 ± 0.2		72.4 ± 0.2		89.2 ± 0.5	
	100	68.3 ± 0.5		67.3 ± 0.4		89.1 ± 0.4	
	200	62.2 ± 0.5		58.3 ± 0.4		88.3 ± 0.2	
	250	54.7 ± 0.3		52.6 ± 0.3		87.8 ± 0.3	

All values are expressed as mean ± SEM (*n* = 3).

**Table 3:** Table showing the binding affinity of all the compounds used in docking.

S.N.	Name of the Ligand	Affinity (kcal/mol)
1	Hydroxytamoxifen	−9.7
2	Caryophyllene	−8.4
3	Caryophyllene oxide	−8.4
4	Humulene	−8.3
5	14-Hydroxy-9-epi-(E)-caryophyllene	−8.2
6	Calamenene	−8.0
7	Muurola-4,10(14)-dien-8beta-ol	−7.9
8	Nerolidol	−7.9
9	Copaborneol	−7.8
10	Copaane	−7.7
11	Linalyl anthranilate	−7.2
12	Eucalyptol	−6.3
13	Terpineol	−6.2
14	Limonenel	−6.1
15	(E)-beta-ocimene	−5.4
16	(Z)-beta-ocimene	−5.3
17	Myrcene	−5.1

**Table 4:** Ligands and the amino acid responsible for the interaction and the type of interactions present. The amino acid involved in van der Waal’s interaction is not given in the table.

S.N.	Name of Ligands	Amino Acids Responsible for Interaction	Types of Interaction
1	Caryophyllene	Leu346	van der Waals, Alkyl
2	Caryophyllene oxide	Leu346	van der Waals, Alkyl
3	Humulene	Leu346	van der Waals, Alkyl
4	14-Hydroxy-9-epi-(E)-caryophyllene	Leu346, Leu384, Leu387	van der Waals, Alkyl, Conventional hydrogen bond
5	Calamenene	Leu346, Leu384, Leu349, Ala350, Trp383, Leu525	van der Waals, Pi-sigma, Pi-alkyl, alkyl

**Table 5:** Complementarity parameters of docked and experimental (3ERT) “enzyme–ligand” complexes.

Ligand	$a_{\mathrm{CF}1}$-Coefficient	$b_{\mathrm{CF}1}$-Coefficient	Rcor2	Sigma	Npoints	MIN (SUMRLRE)	MAX (CF1)
calamenene (model 18)	6.26	−4.003	0.840	0.24	835	3.304	−6.776
Muurola-4,10(14)-diene-1-1-beta-ol (model 3)	6.396	−4.031	0.918	0.34	5650	2.742	−4.184
3ERT	9.041	−4.820	0.972	0.29	5582	2.420	−2.570

**Table 6:** Table showing the parameters for druglikeness of the selected ligands.

Name of the Molecule	Molecular Weight (gm/mol)	Lipophilicity (MLOGP)	H-Bond Acceptor	H-Bond Donor	Molar Refractivity	Drug Likeness
Caryophyllene	204.35	4.63	0	0	68.78	Yes; 1 violation: MLOGP > 4.15
Caryophyllene oxide	220.35	3.67	1	0	68.27	Yes; 0 violation
Humulene	204.35	4.53	0	0	70.42	Yes; 1 violation: MLOGP > 4.15
14-Hydroxy-9-epi-(E)-caryophyllene	220.35	3.56	1	1	69.94	Yes; 0 violation
Calamenene	202.34	5.45	0	0	68.07	Yes; 1 violation: MLOGP > 4.15

## Data Availability

All the data generated and analyzed can be found within the article.
